# IR Spectroscopy: From Experimental Spectra to High-Resolution Structural Analysis by Integrating Simulations and Machine Learning

**DOI:** 10.1021/acs.jpcb.5c04866

**Published:** 2025-10-29

**Authors:** Marvin Scherlo, Dominic Phillips, Ricarda Künne, Emiliano Ippoliti, Klaus Gerwert, Carsten Kötting, Paolo Carloni, Antonia S. J. S. Mey, Till Rudack

**Affiliations:** † Center for Protein Diagnostics (PRODI), Biospectroscopy, 9142 Ruhr University Bochum, Bochum 44801, Germany; ‡ Biomolecular Simulations and Theoretical Biophysics Group, Faculty of Biology and Biotechnology, Ruhr University Bochum, Bochum 44801, Germany; § School of Informatics and Maxwell Institute for the Mathematical Sciences, 151022 University of Edinburgh, Edinburgh EH8 9BT, U.K.; ∥ Department of Biophysics, Ruhr University Bochum, Bochum 44801, Germany; ⊥ Computational Biomedicine, Institute for Neuroscience and Medicine inm-9, 196554 Forschungszentrum Jülich GmbH, Jülich 52428, Germany; # EaStCHEM School of Chemistry, 3124 University of Edinburgh, David Brewster Road, Edinburgh EH9 3FJ, U.K.; ∇ Structural Bioinformatics Group, Regensburg Center for Ultrafast Nanoscopy, 9147 University of Regensburg, Regensburg 93053, Germany; ○ Structural Bioinformatics Group, Regensburg Center for Biochemistry, University of Regensburg, Regensburg 93053, Germany

## Abstract

Understanding biomolecular function at the atomic scale requires detailed insight into the structural changes underlying dynamic processes. Vibrational infrared (IR) spectroscopy—when paired with biomolecular simulations and quantum-chemical calculations—determines bond length variations on the order of 0.01 Å, providing insights into these structural changes. Here, we address the forward problem in IR spectroscopy: predicting high-accuracy vibrational spectra from known molecular structures identified by biomolecular simulations. Solving this problem lays the groundwork for the inverse problem: inferring structural ensembles directly from experimental IR spectra. We evaluate two computational approaches, normal-mode analysis and Fourier-transformed dipole autocorrelation, against experimental IR spectra of *N*-methylacetamide, a prototypical model for peptide bond vibrations. Spectra are derived from simulation models at multiple levels of theory, including hybrid quantum mechanics/molecular mechanics, machine-learned, and classical molecular mechanics approaches. Our results highlight the capabilities and limitations of current theoretical biophysical approaches to decode structural information from experimental vibrational spectroscopy data. These insights underscore the potential of future artificial intelligence (AI)-enhanced models to enable direct IR-based structure determination. For example, resolving the so-far experimentally inaccessible structures of toxic oligomers involved in neurodegenerative diseases, enabling improved disease diagnostics and targeted therapies.

## Graphical abstract

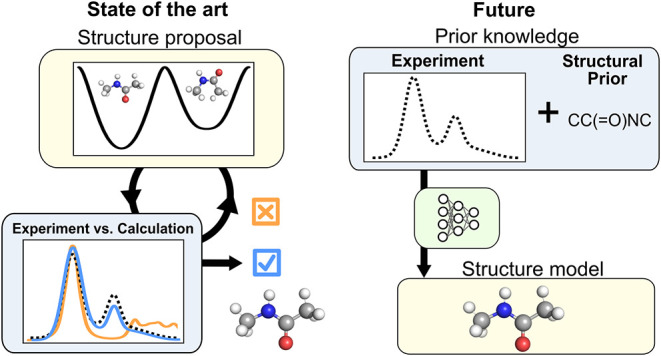

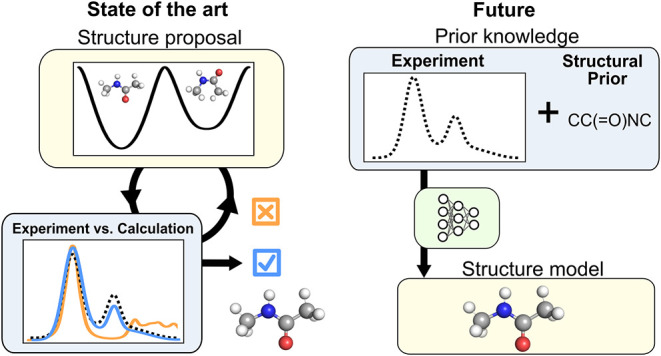

## Introduction

Conformational changes in proteins and biomolecules play a central role in many cellular processes. For many years, spectroscopic methods have been invaluable tools for elucidating the underlying structures and dynamics. While techniques such as Nuclear Magnetic Resonance (NMR) spectroscopy yield atomic-level structures,^1^ and others such as Förster Resonance Energy Transfer (FRET)^2^ and Circular Dichroism (CD)^3^ probe distances and secondary structures, respectively, infrared (IR) spectroscopy, stands out for its combination of sensitivity and temporal resolution.^4,5^

The power of IR stems from its ability to resolve minute structural changes, where a ∼1 cm^–1^ frequency shift corresponds to a change in bond length of ∼0.001 Å.^5^ While initially used for the qualitative and quantitative analysis of organic compounds,^6^ IR’s high resolution also makes it ideal for studying enzyme mechanisms and active site dynamics.^7^ By probing the amide I band, IR spectroscopy distinguishes regions of α-helices and β-sheets, and this has led to its use in biosensors for diagnosing proteinopathies such as Alzheimer’s or Parkinson’s disease.^8,9^ Furthermore, time-resolved Fourier Transform Infrared Spectroscopy (FTIR) offers the unique advantage of being able to capture dynamics on the nanosecond-to-second timescale.^10–12^

However, this remarkable sensitivity creates a significant challenge: the rich information about structure–function relationships is hidden within broad, overlapping vibrational bands that are difficult to assign. Unlike NMR, where spin–spin couplings yield well-defined patterns, IR spectra are more challenging to decode. 2D-IR distinguishes some mode couplings, but it requires femtosecond lasers and suffers from low signal-to-noise ratio.^13^ Therefore, computational biophysics, especially biomolecular simulations and quantum-mechanical calculations, is essential to translate spectroscopic observables into detailed structural models and mechanistic insights. Historically pioneering work has been done in the context of NMR spectroscopy, bridging experiment and theory, by Prof. Peter A. Kollman and collaborators. In this spirit we dedicate this article.^14–20^

Historically, computational approaches have focused on solving the *forward problem*: predicting an IR spectrum from a proposed structure (Figure 1A). A human-in-the-loop procedure then iteratively refines the structure until the calculated spectrum matches the experimental one. To obtain a spectrum, two parts are required: a simulation model, which computes trajectories of structural ensembles, and a spectrum model, which computes spectra from the structural data. Depending on the required accuracy, various molecular dynamics (MD) approaches can be employed, such as *ab initio* quantum mechanics (QM), hybrid quantum mechanics/molecular mechanics (QM/MM) simulations, or classical molecular mechanics (MM). Then, to obtain the spectrum, two primary techniques exist. The first, Normal Mode Analysis (NMA), calculates vibrational frequencies from the Hessian matrix of a single equilibrium structure. Warshel’s,^21^ as well as Tavan and Schulten’s work on the retinal structures^22,23^ validated this approach by obtaining similar IR spectra to experiments. While computationally efficient in its basic form, this approach fails to capture the conformational diversity of molecules at room temperature. This limitation is partly addressed by applying NMA to an ensemble of structures derived from MD simulations either MM or QM/MM.^24–26^ However, this ensemble-based NMA becomes significantly more computationally demanding, as the Hessian matrix must be calculated and diagonalized for a large number of structures. Another approach, typically less computationally demanding, is to compute IR spectra directly from MD simulations via the Fourier transform (FT) of the dipole autocorrelation function^27–31^ which naturally includes the effects of conformational heterogeneity. However, assigning these IR bands to specific nuclear motions is highly challenging compared to the straightforward approach in Normal Mode Analysis. Determining isotopic effects within a dipole autocorrelation-framework requires repeating the underlying MD because the isotopic substitution changes the atomic masses and therefore the dynamics/time-correlation functions, meaning this method typically yields a single, unassigned spectrum, although techniques have been suggested that partially overcome this limitation.^32–34^ By contrast for NMA isotopic substitution is straightforward: updating the masses in the (mass-weighted) Hessian shifts the frequencies without requiring geometry reoptimization, since the conformational minima remain the same.

**Figure 1:**
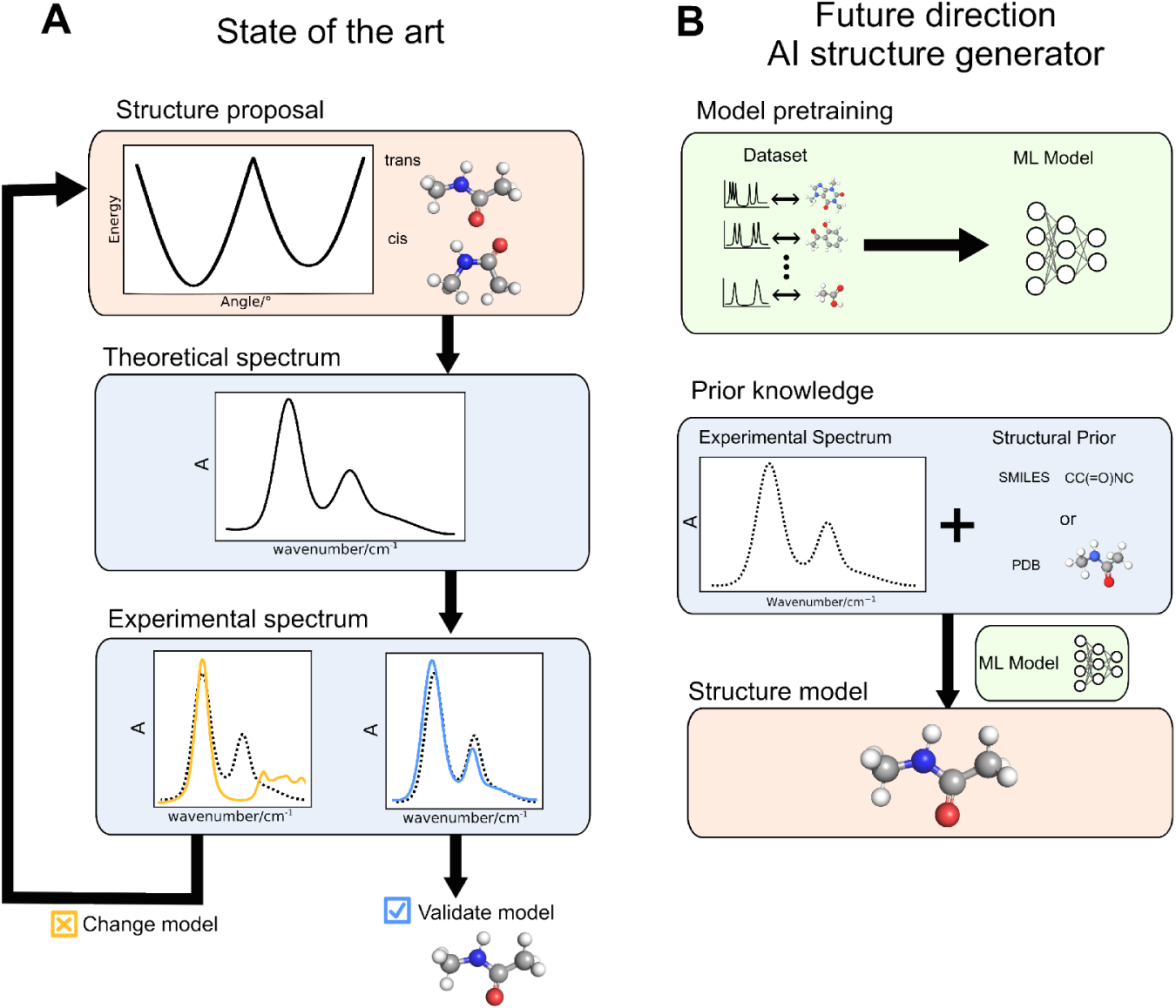
Traditional and ML-enhanced structural spectroscopy strategies. The traditional strategy (A), addressing the forward problem in IR spectroscopy, starts with a structural proposal and a human-in-the-loop iteratively updates the structure by comparing its theoretical spectrum with an experimental reference, until close alignment. The ML-enhanced strategy tackling the inverse problem (B) requires first training a model on a paired spectrum-structure dataset. The experimental spectrum, often conditioned with a structural prior (such as a SMILES string or reference geometry), is then passed as input to the trained model. The model output is a direct prediction of the molecule’s 3D structure based on the experimental spectrum. Other paradigms for ML-enhanced workflows are also possible; see the main text for details.

Complementary to direct first-principles routes (NMA and dipole moment autocorrelation analysis (DMA)), the field has developed spectroscopic frequency maps that relate local electrostatic descriptors (e.g., electric field, field gradient, and geometry) to amide I frequencies and transition-dipole moments. These maps are commonly calibrated on model systems such as *N*-methylacetamide and then transferred to peptides and proteins, enabling exciton-based simulations of 1D- and 2D-IR spectra. For overviews and representative developments, see previous studies.^35–42^

Increasingly, machine learning (ML) methods are providing new ways of accelerating the forward problem. For instance, ML-force fields and dipole models can be trained on density functional theory (DFT) data, enabling MD simulations at a level approaching DFT accuracy but at a fraction of the computational cost, which can then be used with NMA or FT analysis to generate the spectrum.^43^ While accurate, this simulation-based approach is not scalable, as it requires fresh computation for each new molecule. An alternative approach uses supervised ML models to directly map molecular structures to their IR spectra. While early work focused on specific bands like the amide I frequency in proteins,^44^ recent graph neural networks generate full spectra.^45,46^ These models show high correlation (Spearman ∼ 0.9) with ground truth data, but their performance drops significantly for molecules with novel structural features outside of the training data, and there remains substantial room for improvement in predicting absorption intensities and bandwidths.

In addition to accelerating the forward problem, ML techniques are starting to introduce a paradigm shift by attempting to directly solve the *inverse problem*: predicting a molecular structure directly from its spectrum (Figure 1B). This strategy requires training on paired structure-spectrum data, where the structure also encodes all the determining environmental factors, such as solvation, hydrogen-bond networks, ion concentration and protonation states/pH. In practice, pairs can be assembled by combining experimentally validated conformational ensembles (MD simulations with explicit solvent and ions, cross-checked by NMR/SAXS/CD) with matched IR/2D-IR spectra under identical conditions, additionally employing isotope labeling to generate site-specific information. Although predicting a full 3D structure from an IR spectrum alone is currently an unsolved challenge,^47^ significant progress has been made on simplified tasks like predicting functional groups^48^ or generating molecular graphs.^49^ Accuracy is further improved by incorporating prior knowledge or complementary data, with the integration of NMR spectra proving particularly effective.^50^

Here, we explore how machine learning (ML) can advance IR spectroscopy into a method for analyzing molecular structure and dynamics, comparable to NMR spectroscopy. To assess this potential, we compare six theoretical workflows for generating IR spectra, combining two calculation methods (Normal Mode Analysis and Dipole Autocorrelation) with three simulation approaches (Classical MM, QM/MM, and ML-based potentials) hereafter referred to collectively as MD simulations.

We apply these workflows to *N*-methylacetamide, a common benchmark molecule that models a peptide backbone and exists in two distinct conformations, *trans* and *cis*.^51–56^ By validating the calculated spectra against experimental data, we provide a basis for discussing the merits of each simulation and calculation method.

Ultimately, we anticipate that this rigorous integration of experimental and theoretical IR spectroscopy has the potential to become a cornerstone of 4D structural biology, complementing time-resolved X-ray crystallography and cryo-EM. Beyond its broad applicability, this approach holds particular promise for resolving challenging targets such as the heterogeneous aggregates implicated in neurodegenerative diseases such as Alzheimer’s and Parkinson’s. The detailed structural insight gained could lay the foundation for advances in diagnostics and targeted therapies—an achievement that has so far remained out of reach for existing structural biology methods.

## Methods

To account for dynamic ensembles found in IR spectra, we make use of biomolecular simulation strategies to generate these ensembles. We compare different simulation approaches using quantum chemical calculations and molecular dynamics (MD) simulations to assess their ability to produce high-quality theoretical IR spectra.

The overall workflow is presented in Figure 2 and encompasses the following steps. First, a classical molecular mechanics (MM) simulation was used to equilibrate a solvated starting structure of *trans*- and *cis*-*N*-methylacetamide (*cis*-NMA), followed by three different types of biomolecular simulation production runs from which IR spectra are derived. In the following, we present the details of the different methods used to simulate the structural ensemble and to extract the calculated theoretical IR spectra.

**Figure 2:**
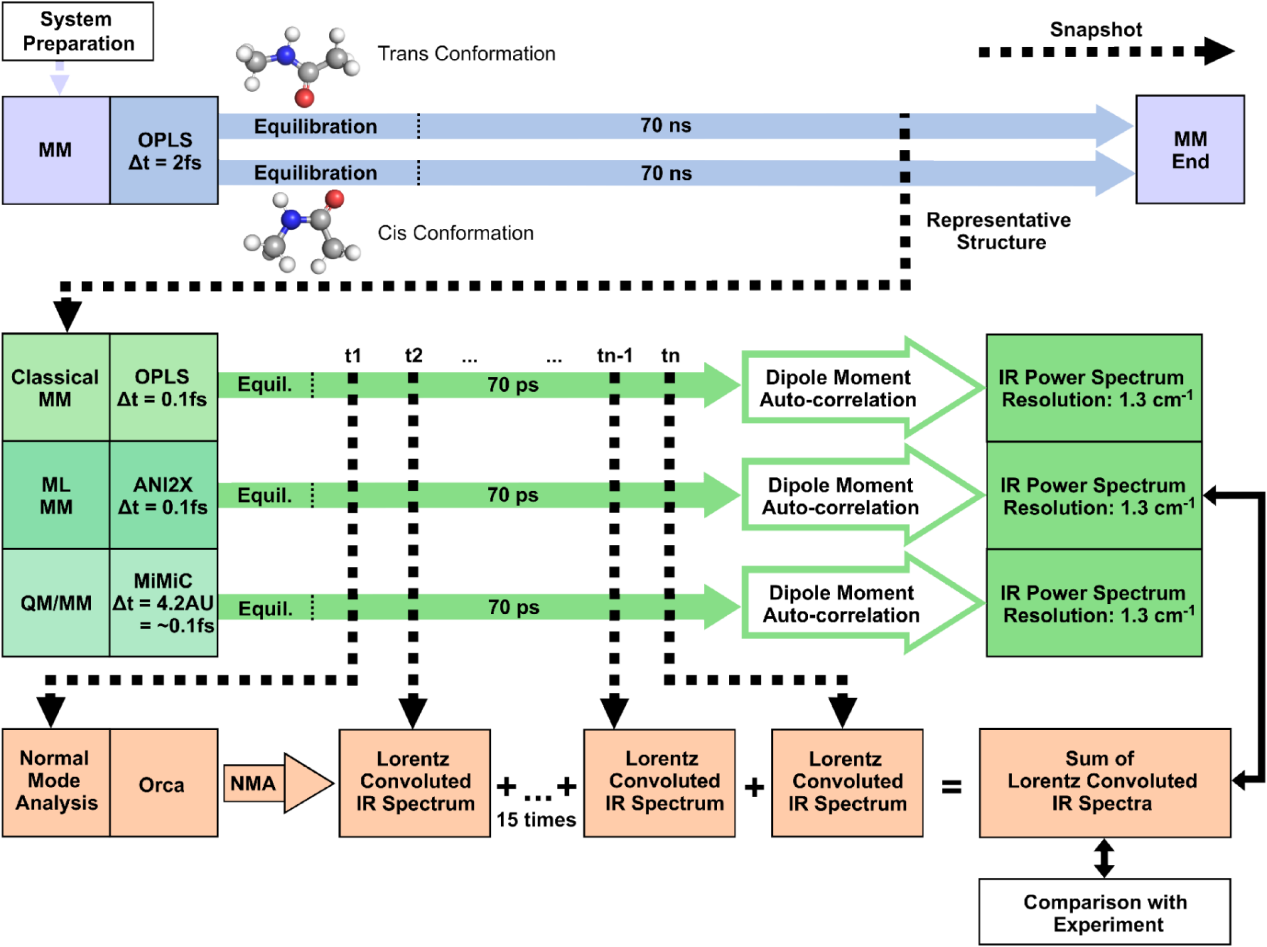
Traditional theoretical IR spectroscopy workflow. First, we performed 70 ns classical molecular mechanics (MM) simulations with a 2 fs step size, to equilibrate the *trans* and *cis* conformations of *N*-methylacetamide in solution. We then used the geometries of the representative simulation structure as starting points for the three simulations sampling the atomic motion within one conformation with a step size of 0.1 fs: A classical MM simulation with the OPLS/AA force field,^57^ a machine-learned potential-based MD simulation with ANI-2x^58^ and a QM/MM simulation using MiMiC.^59,60^ From each of these runs, a theoretical IR spectrum was calculated using both NMA and DMA. For validation, these spectra were compared with the experimental IR spectrum. This workflow allows a systematic evaluation of how both the IR calculation method and the underlying simulation technique affect spectral accuracy and conformational differentiation.

### Simulation System Preparation

The workflow is initiated by preparing the structures of *trans*-NMA and *cis*-NMA (Figure 2, top left corner) using the software packages MAXIMOBY (v. 2025)^61^ and GROMACS (v. 2024.1).^62,63^ Initially, the first and second solvation shells were added using the solvation approach implemented in MAXIMOBY that is based on the Vedani algorithm.^64^ To prevent self-interactions due to periodic boundary conditions, the solvated cluster was placed in a cubic simulation cell with a minimum padding distance of 13 Å. This resulted in a cubic box with an edge length of 44 Å for *trans*-NMA and 42 Å for *cis*-NMA. The box was then filled with bulk water containing 2754 and 2549 water molecules, respectively, using the solvation strategy implemented in GROMACS. To resolve steric clashes in the transition between the second solvation shell and the bulk water, an energy optimization of water hydrogen atoms was performed in MAXIMOBY using the implemented Amber84 ^65^ united atom force field and the force field corresponding TIP3P water model.^66^ Finally, we convert the TIP3P water model to TIP4P.^66^ TIP4P is commonly paired with the Optimized Potentials for Liquid Simulations/All Atom (OPLS/AA) force field^57^ and shows improved dynamic properties. Therefore, it is used for the subsequent simulations. It shows better agreement with experimental measurements for dynamic properties.^66^

### Molecular Mechanics (MM) Simulations

The prepared structure serves as the starting point for the two initial *trans*-*N*-methylacetamide (*trans*-NMA) and *cis*-*N*-methylacetamide (*cis*-NMA) MM simulations, illustrated in Figure 2 as two broad, blue arrows. The systems were heated to 293 K during a 1 ns NVT equilibration with a step size of 1 fs. The temperature was controlled using a velocity-rescaling thermostat^67^ with a coupling constant of 0.1 ps. The heating process was carried out in two stages: Initially, the temperature was gradually increased from 0 to 100 K over the first 100 ps, followed by a further increase to 293 K over the subsequent 900 ps. This temperature was chosen to match the room temperature during the experimental measurements. The system was then equilibrated using an additional 1 ns NVT simulation (step size 1 fs) with a constant temperature of 293 K, followed by a 10 ns NPT simulation (step size 1 fs). The temperature coupling is done with the velocity-rescaling thermostat (coupling constant 0.1 ps) to stabilize the system temperature, and the pressure coupling using a Berendsen barostat^68^ (coupling constant 0.1 ps) to rapidly relax the density to the target. These schemes are well suited for equilibration but do not generate a rigorously correct NPT. Subsequently, a 70 ns NPT production run with a step size of 2 fs was performed, using the Nosé-Hoover thermostat^69,70^ (coupling constant 0.5 ps) and the Parrinello–Rahman barostat^71^ (coupling constant 2.5 ps) which together ensure a physically correct representation of the NPT ensemble. Coordinates and velocities were handed over without reinitialization. Standard observables (T, P) and the peptide bond dihedral ω displayed no discontinuations. To allow for a step size of 2 fs, the heavy atom-hydrogen bonds were constrained with the LINCS algorithm.^72^ We extracted a representative structure (see Figure 3) from each of the *trans* and *cis*-NMA production runs to initiate the more detailed MD simulations. The final MM runs (Figure 2, top filled green arrow) were used to extract spectra with NMA and DMA apply the same simulation strategies using a time step of 0.1 fs and no constraints on any bonds. All run parameter mdp files and the resulting representative MD simulations structures are available at doi: 10.5283/EPUB.77953.

**Figure 3:**
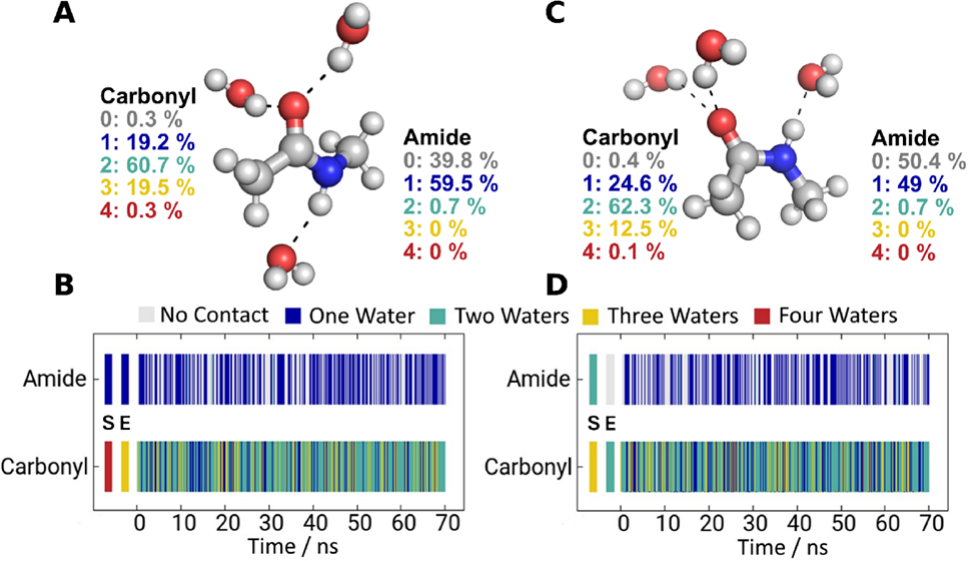
Water interactions with *N*-methylacetamide during a 70 ns (2 fs step size) molecular dynamics simulation. Representative structures for the (A) *trans*- and (C) *cis*-conformations are shown with direct interacting water molecules; bulk solvent is omitted for clarity. The number of water molecules bound to the carbonyl and amide groups was tracked over the simulation time. On average, two water molecules are bound to the carbonyl and one to the amide for both conformations. The percentage of occupation of different numbers of water molecules over the simulation time is shown next to the structures. Panels (B) and (D) show the contact analysis over time, color-coded for the number of different water molecules, for *trans-* and *cis*-conformations, respectively. The first and second data points, labeled S and E correspond to the starting structure and the structure after heating and equilibration.

### Molecular Mechanics (MM) Simulation Analysis

Intermolecule interactions between *N*-methylacetamide and water molecules were analyzed using PyContact14 ^73^ and the contact matrix algorithm implemented in MAXIMOBY.^61^ *N*-methylacetamide possesses no major internal degrees of freedom apart from the *trans*/*cis* isomerization. The representative structure of the first MM MD simulation was therefore defined by its contacts with the surrounding water molecules. We identified the number of water molecules forming a hydrogen bond to each functionality of *N*-methylacetamide every 0.1 ns. On average, about two waters are present at the carbonyl and 1 water at the amide (Figure 3). This was true for both conformations. As there were many structures fulfilling these criteria, we picked the last geometries of the respective simulations doing so and used them as starting structures for the finer MD runs. In Figure 2, these simulation runs are shown as green, broad arrows, originating from green boxes. The three simulation types are listed vertically. All run parameter mdp files are available at doi: 10.5283/EPUB.77953.

### Molecular Dynamics Simulations Using Machine-Learned Interatomic Potentials

Hybrid ML/MD simulations (Figure 2, middle filled green arrow) were initiated by the two representative *N*-methylacetamide structures using for each two types of machine-learned interatomic potentials: ANI-2x and MACE-OFF23 (small).^58,74^ ANI-2x, which currently supports C, O, H, N, F, Cl, and S atoms, was trained on 8.9 million molecular conformations of drug-like molecules from GDB-11 and ChEMBL.^75,76^ The potential is constructed from atomic environment vectors^77^ and trained to reproduce molecular energies and forces at the ωB97*X*/6-31G­(d) level of DFT theory. MACE-OFF23 supports C, O, H, N, F, Cl, S, P, Br, and I atoms and is trained on the SPICE dataset^78^ with an equivariant MACE architecture neural network.^79^ MACE-OFF was trained to reproduce the energies and forces computed at the ωB97M-D3­(BJ)/def2-TZVPPD level of DFT theory.

In all simulations, the ML potential (either ANI-2x or MACE-OFF23) was used to model forces between atoms of *N*-methylacetamide. The molecule was solvated in a water box of classical TIP4P.^66^ All forces between the water and *N*-methylacetamide were modeled with the OPLS/AA force field.^57^ Simulations were performed in OpenMM 8.2 in the NVT ensemble using the recommended BAOAB-Langevin integrator at 293 K (Langevin thermostat) with a friction coefficient of 1 ps.^80^ The equilibration run was 15 ps with a 1 fs time step. The production run was 70 ps with a 0.1 fs time step. Comparable simulation lengths have been used in prior IR studies of *N*-methylacetamide.^81,82^ Inakollu et al. computed FT-DAC spectra for deprotonated serine from a 32 ps production run of a classical MD simulation.^81^ By contrast Schwörer et al. employed QM/MM runs of 100 ps, while discarding the first 15 ps,^82^ further illustrating that our production windows are standard practice. Moreover *N*-methylacetamide is a low-complexity system (12 atoms with 30 vibrational modes) dominated by stretching and bending vibrations. The only slow, large-amplitude motion (trans ↔ cis around ω) is omitted in our simulation setup, as we ran two separate simulations of the *cis* and *trans* conformation. Consequently, velocities and dipole autocorrelation converge on ps timescales. The short 15 ps equilibration suffices to converge the velocity distribution and the 70 ps production segment spans a multitude of correlation lengths providing a nominal resolution of 0.48 cm^–1^. Code for reproducing these experiments is available at https://github.com/dominicp6/mlmd.

### Hybrid Quantum Mechanics/Molecular Mechanics (QM/MM) Simulations

QM/MM calculations (Figure 2, bottom filled green arrow) were performed through the MiMiC interface.^59,60^ MiMiC is a framework to perform multiscale simulations in which loosely coupled external programs describe individual subsystems at different resolutions and levels of theory, particularly suitable for HPC setups.^83^ In this work, MiMiC coupled the DFT-based quantum code CPMD^84^ with the popular classical molecular dynamics code GROMACS.^62,63^ To ensure method comparability, we adopted a solute-only QM region in the QM/MM simulation: *N*-methylacetamide was treated at the QM level, while all water molecules were described identically at the MM level in all protocols (MM, ML, and QM/MM with electrostatic embedding). Holding the solvent model fixed means that only the solute description changes between methods, enabling a direct comparison. Consequently, any differences in the resulting spectra are attributed to the methodology rather than to variations in environmental treatment. Additionally, hydrogen-bond partners exchange on the ps timescale and would require an adaptive QM region within the QM/MM MD which is not implemented in the here used software packages. The QM regions were treated at DFT level of theory with the PBE recipe for the exchange-correlation functional.^85^ The wave function of the QM region was expanded in a plane-wave basis set up to an energy cutoff of 70 Ry. Only valence electrons were explicitly treated, while core electrons were described using norm-conserving pseudopotentials of the Martins–Troullier type.^86^ The MM water molecules were described by the TIP4P model^66^ and the OPLS/AA force field.^57^

### Dipole Moment Autocorrelation Analysis (DMA)

One method for calculating IR spectra is based on the autocorrelation analysis of the dipole moment. This approach takes advantage of a key principle of infrared spectroscopy: IR light is only absorbed if the molecular vibrations lead to a change in the dipole moment. The dipole moment autocorrelation function captures these fluctuations by monitoring how the dipole moment evolves over time during a molecular dynamics simulation with time steps of 0.1 fs. The DMA is illustrated in Figure 2 following the path of the three MD simulations and resulting in the IR Power Spectrum (green boxes on the right). For QM/MM, the dipole moment is calculated by CPMD for every step. For MM and ML trajectories, it is computed from OPLS/AA fixed charges (the ML model provides only intramolecular terms, nonbonded interactions and charges are from OPLS/AA).^85^ To ensure that relevant vibrational modes are sampled adequately, the simulation must run long enough to allow multiple oscillation cycles. The resulting IR power spectrum is obtained by applying a fast Fourier transform (FFT) to the autocorrelation data and subsequently corrected using a quantum correction factor. In molecular dynamics simulations—including QM/MM and CPMD, the motion of atoms is treated classically. As a result, quantum effects in vibrations, especially in fast bond oscillations, are not fully captured. To account for quantum mechanical effects during the MD simulation, we multiplied the IR intensities of the FT-DAC spectrum by the standard quantum correction factor,

$$Q\left(\overset{∼}{\nu }\right)=\frac{x}{1-e^{-x}},x=\frac{hc\overset{∼}{\nu }}{k_{B}T}\approx \frac{1.4388K\cdot \mathrm{cm}}{T}\cdot \overset{∼}{\nu }$$

depending on the temperature T and wavenumber *ν̃*.^87,88^ This gives the correct temperature dependence for harmonic vibrations. The here used method provides a spectral resolution of 0.48 cm^–1^, but does not allow a straightforward assignment of individual normal modes.

### Normal Mode Analysis

An alternative method to compute IR spectra is Normal Mode Analysis (NMA). To calculate IR spectra, we performed NMA calculations initiated by structures from each of the three different detailed simulation trajectories (each 70 ps) with 5 ps distance (15 structures). These structures are depicted in Figure 2 as black dotted arrows originating from the three MD simulations. We used the QM/MM embedded NMA frequency calculation method implemented in ORCA 5.0.4.^89^ This scheme differentiates between a high layer, consisting of the area that is treated quantum mechanically, and a low layer, consisting of the area that is treated using classical molecular mechanics. The QM part contained the *N*-methylacetamide and the closest 10 water molecules. We tested varying numbers of QM-treated water molecules. A lower amount yielded a worse experimental agreement, while a higher amount did not improve it and increased computation time. The MM part contained all remaining solvent molecules. The eigenvalues and eigenvectors of the normal vibrations are obtained by diagonalizing the Hessian. One important condition for this calculation is that the structure is in an energetic minimum. To reach a minimum structure we performed an iterative QM/MM and MM optimization in the same way as described by Mann et al.^90^ but replaced the QM software Gaussian by ORCA. Briefly, the partial charges of the QM region are first calculated by ORCA using the QM/MM approach with electrostatic embedding and handed over to MAXIMOBY for the MM optimizations. Then three alternating optimization cycles are performed: In each cycle, MAXIMOBY optimized only the MM subsystem (QM coordinates fixed), followed by an ORCA 5.0.4 QM/MM optimization of only the QM subsystem (MM coordinates fixed).^61^ The coordinates and charges from each step were passed on to the respective following step. The calculation of the charges in the QM region derived from the electrostatic potential follows the Merz–Singh–Kollman (MK) scheme. The MM optimizations were performed using the Amber5 force field.^91^ The QM calculations were performed using the functional PBE with the basis set 6-31G*. This turned out to be the most economic functional and basis set combination to calculate IR spectra as shown by an extensive comparison study.^90^ The calculated intensities were fitted with a Lorentzian function with a width of 20 wavenumbers and subsequently summed up over the 15 snapshots to generate the final spectrum, illustrated in the orange row in Figure 2.

### Experimental Infrared Spectroscopy (ATR-FTIR Measurements)

ATR-FTIR measurements of *N*-methylacetamide in H_2_O were performed using a Vertex-70-spectrometer (Bruker Optik, Ettlingen, Germany) in the double-sided, forward–backward mode. The spectral resolution was 2 cm^–1^ and the scanner velocity 16 kHz. The resulting interferograms were processed using the Mertz phase correction, a Blackman–Harris three-term apodization function and a zero filling factor of 4. The ATR-accessory integrated in the spectrometer was the DuraSampl*IR* II (Smiths Detection, London, England) with nine active reflections. For the spectra, an average of 224 scans was used. The background spectra included 112 scans. *N*-Methylacetamide was obtained from Sigma-Aldrich Chemicals (St. Louis, USA).

## Results and Discussion

Our results and discussion are split into two parts; in the first part, we evaluate different approaches following the traditional strategy of connecting IR spectroscopic data with structural models addressing the forward problem of theoretical IR spectroscopy. In the second part, we discuss how the evaluated approaches pave the way for solving the inverse problem of assigning structures to spectra directly and the potential to advance IR spectroscopy to a direct structure-giving method with sub-Ångström resolution.

### Sampling of the Conformational Energy Landscape Results in Two Stable Solvated *N*-Methylacetamide Conformations

*N*-Methylacetamide is a well-studied^51–56^ benchmark system to model a peptide bond. For IR spectroscopy to be useful as a structural interrogation method, such benchmark systems are essential to elucidate more complex protein structures in the future. To compare our experimental IR spectrum of *N*-methylacetamide to the different theoretical methods, we need a robust understanding of the conformational space of *N*-methylacetamide, which we here generated from molecular dynamics simulations. *N*-methylacetamide has two dominant conformers *cis*-*N*-methylacetamide (*cis*-NMA) and *trans*-*N*-methylacetamide (*trans*-NMA), with the *trans* form being 2.5 kcal/mol more stable than the *cis* form.^92–95^ Dynamic NMR reports the interconversion free energy for *N*-methylacetamide Δ*G*_*trans*→*cis*_ as 21.3 kcal/mol and Δ*G*_*cis*→*trans*_ as 18.8 kcal/mol.^96^ Eyring estimates at 293 K place the *trans* ↔ *cis* rates on the seconds to minutes timescales, far beyond our ps-μs MD windows. Accordingly, no interconversion is expected under our conditions. Consistent with this, 1D potential energy scans (PBE/6-31G* and OPLS/AA) of the peptide torsion ω give Δ*E*_*trans*→*cis*_/Δ*E*_*cis*→*trans*_: 21.3/18.3 and 20.6/16.4 kcal/mol, respectively, which should be regarded as indicative rather than rigorous free energy barriers. As a result, we ran two separate MM simulations of the two conformers to obtain equilibrated, solvated representative structural models of *cis*-NMA and *trans*-NMA (see the blue part of the workflow Figure 2). Classical force fields resolve *cis/trans* amide via torsional and nonbonded terms and typically maintain amide planarity; however, absolute barrier heights depend on the method and the environment. In all cases, at room temperature, the *cis/trans* interconversion of *N*-methylacetamide is far slower than the ns sampling windows employed. Using Eyring Transition State Theory with the calculated potential energy barriers from the ω torsion scan, we estimate timescales of ∼6.3 min from *trans* to *cis* and ∼0.28 s from *cis* to *trans* at 293 K, so conformations initialized in *cis* or trans remain in that state on these timescales. As expected, no interconversion between *cis* and *trans* isomers is observed in the 70 ns MM trajectories. Going beyond systems with high barriers, different conformers can be identified with sufficiently long MM simulations, and their populations estimated from the simulations. However, in our study, we assigned these two conformations *a priori* and used the initial MM simulation to analyze and identify subconformer dynamics and their respective water interactions. The identified representative structures were used to start all production runs for our theoretical IR spectra calculation. The pattern of water molecules forming hydrogen bonds with *N*-methylacetamide is the primary criterion for selecting the representative structure of each conformation (Figure 3A,C).

In the *trans* conformation, two water molecules are hydrogen-bonded to the carbonyl functionality for the majority of the simulation. However, transition states with one or three bound water molecules are frequently observed. The amide has the tendency to form only one hydrogen bond. One water molecule is present in ∼60% of the simulation time, while the remaining ∼40% corresponds to transition states without a clearly defined hydrogen bond. The *cis* conformation shows the same general trend, albeit with a slight shift to a lower average number of hydrogen bonds, while the median remains unchanged. The representative structures used as starting points for the following evaluation of theoretical IR spectroscopy approaches were chosen to reflect these hydrogen bonding patterns and are shown in Figure 3A for *trans* and Figure 3C for *cis*, respectively, while the frame-wise contact analysis is shown in Figure 3B,D.

### Efficient Workflows for Evaluating Traditional Theoretical IR Spectroscopy Enable the Connection between Structural and Spectral Data

We calculated and measured IR spectra of *N*-methylacetamide to evaluate the different strategies that connect IR spectroscopic data with structural models addressing the forward problem (Figure 1A). The experimentally measured spectrum serves as a gold standard to evaluate the calculation strategies. An overview of the IR spectra calculation workflow is illustrated in Figure 2. Calculation parameters and experimental conditions are detailed in the Methods section. The initially determined representative structures of the conformational energy landscape minima are used to investigate the detailed atomic motion within a given *N*-methylacetamide conformation. We performed MD simulations using three different levels of theory: classical molecular mechanics (MM) force field-based, machine-learned potential-based molecular dynamics (ML), and hybrid quantum mechanics/molecular mechanics (QM/MM) simulations. To calculate the IR spectra based on the structural data, we used the two state-of-the-art approaches: normal-mode analysis (NMA) and dipole moment autocorrelation (DMA).

The resulting spectral comparison is presented in Figure 4. For spectral analysis, two key spectral regions were considered: (i) the amide I and II bands (1500–1700 cm^–1^), reflecting the C–O stretching and a combination of C–N stretching and N–H bending vibrations, respectively, and (ii) the fingerprint region (1250–1450 cm^–1^) which includes methyl-bending and amide III modes. The peak positions in these regions are listed in Table 1, while Table 2 summarizes the deviations between calculated and experimental peak positions. Since assigning atom groups to each peak using DMA is highly challenging and controversial, we focus instead on the peak pattern defined by the spectral band positions and intensities. Mode assignments are based on the established vibrational signatures of the relevant functional groups, as also shown by Chen et al.^93^

**Figure 4:**
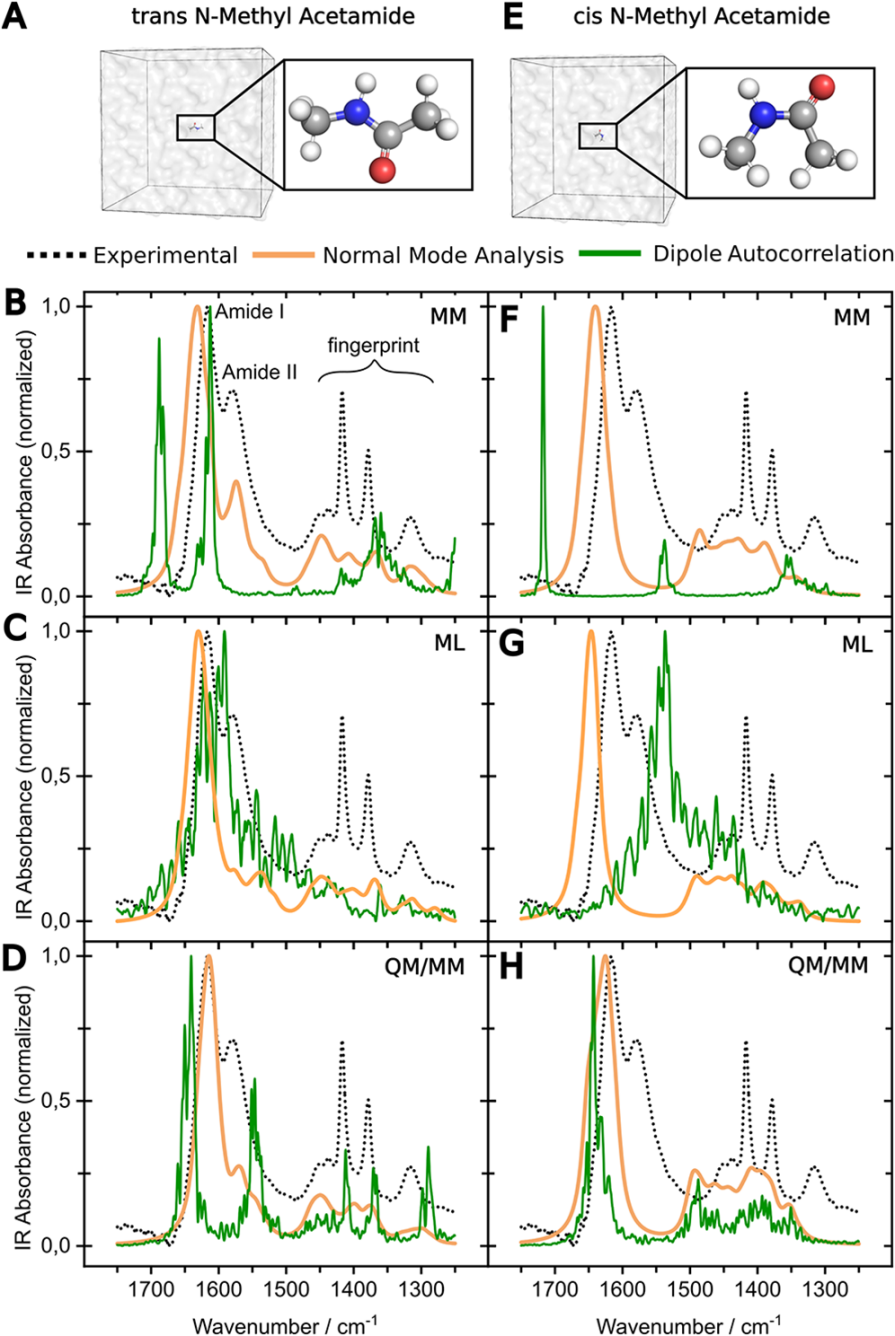
Comparison of theoretical and experimental IR spectra. (A) shows the simulation system for solvated *trans*-NMA and (E) the one for *cis*-NMA. The left column shows the theoretical IR spectra calculated based on NMA (orange) and dipole moment autocorrelation (green) for *trans*-NMA (B–D) and the right one for *cis*-NMA (F–H) compared to the experimental spectrum (black dashed line). Compared are three different methods to obtain the input geometries for the spectra calculation, namely MM (B,F), machine-learned MD (ANI-2x results shown) (C,G), and QM/MM using MiMiC (D,H).

**Table 1:** Comparison of the Calculated and Experimental Peak Positions

Method			Band Assignment/cm^–1^		
Conf.	Force Field	Calculation	Amide I	Amide II	Fingerprint Region
Experiment			1617	1576	1439 1417 1379 1317
*trans*	MM	NMA	1631	1574	1448 1408 1367 1316
		DMA	1688	1613	1418 1368 1360
	ML ANI-2x	NMA	1630	1537	1448 1402 1369 1313
		DMA	1591	-	1064
	ML MACE-OFF	NMA	1624	1532	1449 1410 1374 1321
		DMA	1555	-	1060
	QM/MM	NMA	1614	1570	1449 1399 1376 1300
		DMA	1641	1547	1412 1371 1289 1290
*cis*	MM	NMA	1640	1486	1429 1390 1345
		DMA	1718	1538	1357 1351
	ML ANI-2x	NMA	1647	1490	1458 1339 1391 1340
		DMA	1537	-	1133 1074 1052
	ML MACE-OFF	NMA	1649	1493	1453 1428 1389 1349
		DMA	1498	-	1076 1036
	QM/MM	NMA	1625	1493	1464 1444 1409 1397 1354
		DMA	1643	1488	1394 1350

**Table 2:** Deviation of the Assigned Calculated Peak Positions to the Experimental Ones

Method			Deviation from Experiment/cm^–1^		Absorbance Ratio
Conf.	Force Field	Calculation	Amide I	Amide II	Amide I/Amide II
Experiment			0	0	1.40
*trans*	MM	NMA	+14	–2	2.52
		DMA	+71	+37	0.89
	ML ANI-2x	NMA	+13	–39	5.92
		DMA	–26	-	-
	ML MACE-OFF	NMA	+7	–44	7.14
		DMA	–62	-	-
	QM/MM	NMA	–3	–6	3.63
		DMA	+24	–29	1.79
*cis*	MM	NMA	+23	–90	4.36
		DMA	+101	–38	5.16
	ML ANI-2x	NMA	+30	–86	6.40
		DMA	–80	-	-
	ML MACE-OFF	NMA	+32	–83	6.05
		DMA	–119	-	-
	QM/MM	NMA	+8	–83	3.83
		DMA	+26	–88	4.36

### All Combinations of Theoretical Methods Clearly Distinguish between the *trans* and *cis* Conformations, with All *trans*-NMA Spectra Showing Markedly Better Agreement with the Experimental Data Than Their *cis* Counterparts

All *cis*-NMA spectra calculated by NMA consistently exhibit two bands in the amide region that are separated from each other. While the dominant calculated peak is in the region of the amide I, the second peak is red-shifted to the region around 1485 cm^–1^. This strongly red-shifted second peak is not present in the experiment and represents the key marker band to distinguish between *trans*- and *cis*-NMA. This clear peak separation is also observed in the calculated spectra from DMA based on the MM and QM/MM data. In contrast, the ML-based DMA spectra for both *cis*- and *trans*-NMA show only one dominant peak with a broad shoulder. However, the wavenumber of this maximum peak strongly differs between the conformations allowing us to distinguish them. The *cis*-NMA peak is red-shifted by ∼54 cm^–1^ compared to the one of *trans*-NMA using ANI-2x and ∼57 cm^–1^ using MACE-OFF (Table 1).

By thermodynamic preference, *N*-methylacetamide exists predominantly in the *trans* conformation (98.5%), as *trans*-NMA is 2.5 kcal/mol more stable than *cis*-NMA.^97,98^ Under these thermodynamic considerations, we find that all the calculated spectra for the *trans* conformation agree better with the experiment than the calculated spectra for the *cis* conformation. In systems with more equally distributed conformations under experimental conditions, population-weighted IR spectra need to be calculated using MM simulations or other methods to sample the conformational space. Such weighting has been successfully applied to flexible molecules in solution, demonstrating the importance of thermodynamic sampling in spectral predictions.^99^ In the case of *N*-methylacetamide, however, the overriding dominance of *trans*-NMA (98.5%) makes the need for population-weighting negligible. We consider only the results for *trans*-NMA for our evaluation in the following.

### All *trans*-NMA Spectra Calculated by Normal Mode Analysis Represent the Experimental Features Well, with the Best Agreement for the QM/MM-Based Method

All simulation approaches using NMA as the subsequent spectra calculation method successfully reproduced the main spectral characteristics of *trans*-NMA observed experimentally. These include a prominent amide I band, a detectable amide II band, and characteristic bands in the fingerprint region. However, there are some detailed deviations from the experimental spectrum:

First, for all methods, deviations are observed in the relative band intensities. Specifically, the calculated amide I/II ratio is not in agreement with the experimental data, due to an underestimation of the amide II absorbance. Moreover, the calculated peak around 1450 cm^–1^ is always the most prominent one in the fingerprint region, while it is the weakest in the experimental data. The remaining fingerprint bands are broadened and less intense than experimentally observed.

Second, a detailed analysis of the amide region reveals that for both the MM and the QM/MM-based spectra, the amide I and II peaks are in very good agreement with the experiment. In contrast, the ML-based spectrum does not reflect the experimental pattern as the amide II peak is red-shifted with low absorbance. The spectra of the two ML interatomic potentials ANI-2x and MACE-OFF display an identical general shape and share all major spectral characteristics, with only slight deviations regarding the peak positions. Figure 4 displays the results using ANI-2x. A comparison between the two ML-based potentials is found in Figures S1 and S2.

Third, the number of calculated peaks and their positions within the fingerprint region agree with the experiment for all methods. However, the overall pattern is not reproduced in detail by any of the methods, as the absorbance and band broadening of each of the four peaks deviates from the experiment.

In summary, the MM- and QM/MM-based NMA-calculated peak positions agree with the experimental data, but further refinement of the method is needed to improve the amide I and amide II absorbance ratios and the shape of the fingerprint region. The QM/MM-based result is slightly better than the MM-based one, which in turn is in better agreement than the ML-based result. The very similar results of MM- and QM/MM-based spectra indicate that the OPLS/AA MM force field samples a conformational ensembleclose to the QM/MM one, and QM/MM optimization of snapshots is already sufficient to capture the key spectral features. This holds true for at least simple structures with limited intra-conformational dynamics.

### Examining the Dipole Moment Autocorrelation-Based Calculations, the QM/MM-Derived Spectrum Matches the *trans*-NMA Experimental Features Best

The DMA approach depends even more strongly on the accuracy of the underlying MD simulation approach than the NMA. For DMA, every written structure (all 700 000 frames at 0.1 fs) of the simulation accounts for the calculation, while for NMA, only 15 QM-optimized structures are used. Therefore, the deviation between the three MD methods is stronger than for the NMA-calculated spectra.

Among all DMA-calculated spectra, the experimental overall pattern in the amide region is best described by the ML-based ANI-2x potential spectrum. The high-absorbance peak is red-shifted by 26 cm^–1^ compared to the experiment. However, for the ML-based MACE-OFF potential (Figure S1) the high-absorbance peak is red-shifted by 62 cm^–1^ (Table 2) and broadened relative to the experiment. In contrast, the MM- and QM/MM-based spectra show two distinct, well-separated peaks, not in accordance with the experimental shape. The position of the tentative amide I peak deviates with 26 cm^–1^ in a comparable magnitude to the ANI-2x spectrum, but is blue-shifted in contrast to it. The MM Peak deviates most clearly from the experiment with a blue-shift of 71 cm^–1^. Interestingly, the MM- and QM/MM-based spectra both almost exactly reproduce the amide I and amide II absorbance ratios (Table 2).

The fingerprint region reveals the most pronounced differences between the different approaches. In both ML-based spectra, this region is red-shifted by approximately 300 cm^–1^ and lacks a well-defined peak pattern (Figure S2). The MM-based spectrum reproduces three of the four experimental peaks (Table 1). The QM/MM-based spectrum shows four peaks in close proximity to the four experimental ones and overall exhibits the best agreement with the experiment, although deviations remain.

### Among All Methods, the QM/MM-Based NMA Spectrum Shows the Best Agreement with the Experiment, Although Further Refinement Should Be Considered

The results demonstrate that the choice of simulation method and IR calculation technique strongly influences the agreement with experimental IR spectra and even the ability to distinguish conformers. QM accuracy is needed to reproduce experimental data, as indicated by the best experimental fit of the QM/MM-based spectra among all DMA calculations. However, MM simulations with subsequent QM/MM optimization provides the most economic way to obtain a solid agreement with the experiment compared to the needed computational power. The shifted fingerprint region for the ANI-2x and MACE ML interatomic potential indicates a clear issue with the way the methyl parameters are learned, suggesting that improvements are needed. Nevertheless, we anticipate that ML-based force fields are the future and will advance theoretical IR spectroscopy methods. However, to date, there is still a clear need to improve such force fields to reach QM accuracy with less computational costs.

Among all methods, the best agreement between calculated and experimental spectra is observed for QM/MM-based NMA calculations. Nevertheless, further refinement remains possible, especially for the peak pattern of the fingerprint region. This region is, in fact, best reproduced by the QM/MM-based DMA calculations. A systematic benchmarking of the specific methodological options and implementations across the different software packages may further improve the agreement between the calculated spectra and the experimental data. All in all, the differences in peak positions and shapes across methods reflect the sensitivity of the calculated spectra to the quality of detailed atomic changes. This sensitivity underlines the high potential of IR spectroscopy to advance as a crucial structure-giving method by combining theoretical and experimental IR spectroscopy.

### Reliable Methods for Computational Predicting IR Spectra Pave the Way to Solve the Inverse Problem of Assigning Structures to Spectra Directly

The power of traditional theoretical IR spectroscopy approaches is evident, nevertheless, the applicability of these traditional approaches becomes increasingly constrained as molecular systems grow in size and complexity. In particular, systems involving the explicit consideration of surroundings, such as proteins or condensed-phase environments, pose significant challenges. The computational expense of quantum mechanical calculations scales poorly with system size, and the harmonic approximation inherent in NMA often fails to capture anharmonic effects and environmental influences that are critical in such contexts. Although MD-based approaches incorporate some of these effects, they remain time-consuming, computationally intensive, and require extensive sampling, particularly for large or flexible systems, where achieving sufficient sampling to capture all relevant conformations becomes increasingly challenging. Moreover, the outcomes of MD simulations can strongly depend on the chosen starting structure or the employed parameters, which may limit the exploration of the conformational space.

The ideal instead is, from just an experimental spectrum as input, to determine conformers and vibrational modes that give rise to different parts of the spectrum without biomolecular simulations or other computationally intensive modeling approaches. This is also known as the inverse problem.^47^

While, to date, we cannot reliably solve for this inverse problem, recent advances in machine learning (ML), particularly in deep learning, offer opportunities to streamline the workflow and possibly increase the accuracy of the mapping between conformers and their vibrations, capturing a specific spectrum of interest. For example, ML-derived force fields accelerate dynamical modeling, substituting the role of QM/MM or DFT approaches^100^ are a promising alternative, but still leave room for improvement, as demonstrated by the poor capturing of the methyl vibrations in the fingerprint region of *trans*-NMA. Additionally, ML algorithms automate spectra preprocessing tasks such as denoising, spike removal, baseline correction, and feature extraction.^101–103^ ML also aids in hybrid workflows by assisting in ranking candidate structures prior to further refinement.^104,105^

Progress in the inverse problem itself is ongoing.^47^ Current approaches simplify the problem by predicting molecular graphs or SMILES sequences instead of full 3D structures or focusing on easier tasks such as classifying functional groups. Convolutional neural networks have been employed for functional group prediction using FTIR data,^48^ and models combining IR data with molecular formulas have been used to generate SMILES strings.^49^ Integrating diverse spectral data, particularly NMR, generally outperforms methods relying solely on IR for structure elucidation, although 3D molecular structure prediction still remains a challenge.^50,106–108^ Emerging deep generative models, such as diffusion and flow-matching techniques, show promise in tackling these complex, 3D structural inverse problems.^109^ Flow matching has already been applied to 3D structure elucidation from Raman rotational spectra.^110^

A major challenge is in obtaining suitable datasets for training the models. Common benchmarks such as QM9, which include small molecules up to nine heavy atoms, are often supplemented with simulated spectra which fail to capture the full complexity of real data.^47,103,111^ Another challenge is how to incorporate implicit chemical principles—such as valency rules, ring strain or stereochemical preferences—directly into the learning process. Without these, models can struggle to generalize and scale to larger molecules.^47^ Moreover, while many methods achieve promising top-*k* accuracy (how often the correct structure is found in the *k* most probable predictions), achieving high top-1 accuracy is still difficult.^50^ Although recent literature has explored providing confidence estimates to predictions,^50^ explaining *why* a prediction was made remains an open challenge.

Looking ahead, machine learning (ML) is poised to transform the theoretical analysis of infrared spectra by enabling capabilities that extend beyond the traditional computational methods. As larger and more diverse datasets of experimental and computed spectra become available, ML models will increasingly be able to capture the intricate relationships between molecular structure and vibrational features.

Such capabilities would significantly enhance our ability to interpret spectral data, particularly in cases where traditional approaches struggle, such as in complex mixtures, flexible biomolecules, or materials under dynamic conditions. Ultimately, this may enable automated structure elucidation and spectral interpretation, with potential applications ranging from high-throughput screening to in situ monitoring of chemical processes.

While quantum mechanical methods will continue to provide the theoretical foundation and interpretative depth essential to vibrational spectroscopy, ML-based techniques are expected to play an increasingly central role. With ongoing advances in model architectures, training strategies, and integration with experimental workflows, the coming years are likely to witness a profound shift toward hybrid approaches that combine the strengths of physics-based and data-driven methods for theoretical IR spectroscopy.

## Conclusion

In summary, we demonstrate that current theoretical computational biophysics approaches, particularly MD simulations in combination with QM/MM optimization and normal-mode analysis, accurately reproduce key features of experimental IR spectra and distinguish molecular conformations such as *cis*- and *trans*-NMA. This proves that the combination of experimental and computational IR spectroscopy is capable of mining the structural information encoded in the measured data. The sensitivity of vibrational spectroscopy allows for obtaining structural models with sub-Ångström resolution, limited by the accuracy of the calculation method. However, the required iterative feedback loop between computational structure prediction and experimental validation is time-consuming and computationally costly. It becomes particularly challenging when the method is applied to molecules with multiple conformations or larger proteins. However, solving this challenge is desirable as it will be essential for, e.g., resolving heterogeneous aggregates, such as those implicated in neurodegenerative diseases, which are currently unresolvable by existing structural biology methods. Identifying the structure of such drug targets will assist to improve targeted therapy and diagnostics in future.

Building on recent advances in artificial intelligence tools, the logical next step is to move beyond these forward models and develop methods that directly infer structural information from experimental IR spectra, the so-called inverse problem. We anticipate that machine learning, which has already proven useful in many related fields, will play a crucial role in this endeavor. Its application to this specific problem is still in its infancy with foundational work needed to establish reliable models, generate diverse training data, and incorporate chemical knowledge. Solving the inverse problem will pave the way for advancing IR spectroscopy as a structure-giving method, providing structural and dynamic models with sub-Å resolution even of heterogeneous structures.

## Supplementary Material



## Notes

(Figure S1) IR spectra using MACE ML-force field following the same workflow (NMA and DMA); comparison to ANI-2X ML-force field; (Figure S2) DMA spectra of both ML-force fields at lower wavenumbers (PDF)
