# Supplementary material for: IR Spectroscopy: From Experimental Spectra to High-Resolution Structural Analysis by Integrating Simulations and Machine Learning
Source: J Phys Chem B. 2025 Oct 29;129(45):11652–65. doi: 10.1021/acs.jpcb.5c04866 (PMC12621253; doi:10.1021/acs.jpcb.5c04866)
Supplement: Supplementary file 1 [file jp5c04866_si_001.pdf]

Supporting Information for IR Spectroscopy: from Experimental Spectra to High-Resolution Structural Analysis by Integrating Simulations and Machine Learning

Marvin Scherlo,[†,‡,△] Dominic Phillips,[¶,△] Ricarda Künne,[†,§] Emiliano Ippoliti,[‖] Klaus Gerwert,[†,§] Carsten Kötting,[*,†,§] Paolo Carloni,[*,‖] Antonia S.J.S. Mey,[*,⊥] and Till Rudack[*,#,@,‡]

†*Center for Protein Diagnostics (PRODI), Biospectroscopy, Ruhr University Bochum, 44801 Bochum, Germany*

‡*Biomolecular Simulations and Theoretical Biophysics Group, Faculty of Biology and Biotechnology, Ruhr University Bochum, 44801 Bochum, Germany*

¶*School of Informatics and Maxwell Institute for the Mathematical Sciences, University of Edinburgh, EH8 9BT Edinburgh, UK*

§*Department of Biophysics, Ruhr University Bochum, 44801 Bochum, Germany*

‖*Computational Biomedicine, Institute for Neuroscience and Medicine INM-9, Forschungszentrum Jülich GmbH, 52428 Jülich, Germany*

⊥*EaStCHEM School of Chemistry, University of Edinburgh, David Brewster Road, EH9 3FJ Edinburgh, UK*

#*Structural Bioinformatics Group, Regensburg Center for Biochemistry, Regensburg Center for Ultrafast Nanoscopy, University of Regensburg, 93053 Regensburg, Germany*

@*Structural Bioinformatics Group, Regensburg Center for Biochemistry, University of Regensburg, 93053 Regensburg, Germany*

△*Contributed equally to this work*

E-mail: Carsten.Koetting@ruhr-uni-bochum.de; p.carloni@fz-juelich.de; antonia.mey@ed.ac.uk; till.rudack@ur.de

Supporting Information

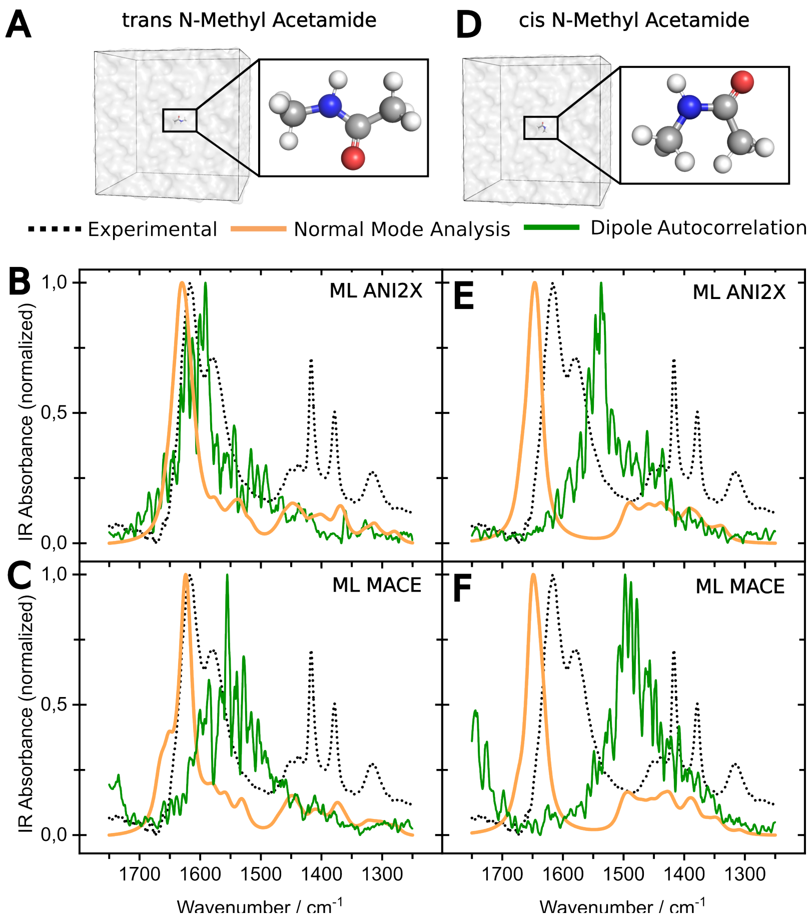

Figure S1: Comparison of theoretical and experimental IR spectra of N-Methylacetamide. **A** shows the simulation system for solvated *trans*-NMA and **D** the one for *cis*-NMA. The left column shows the theoretical IR spectra calculated based on NMA (orange) and dipole moment auto-correlation (green) for *trans*-NMA (**B,C**) and the right one for *cis*-NMA (**E,F**) compared to the experimental spectrum (black dashed line). Compared are two different machine-learned force fields used in a simulation to obtain the input geometries for the spectra calculation, namely ANI2X (**B,E**) and MACE (**C,F**).

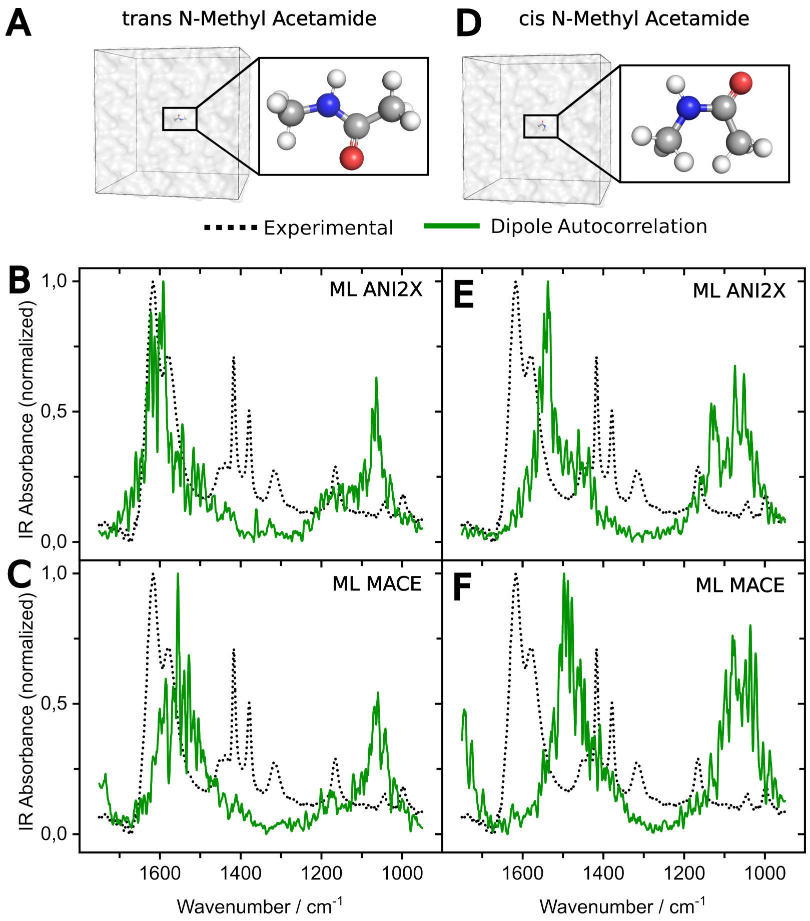

Figure S2: Comparison of theoretical and experimental IR spectra. **A** shows the simulation system for solvated *trans*-NMA and **D** the one for *cis*-NMA. The left column shows the theoretical IR spectra from 950-1750 cm^{-1} calculated based on Dipole Moment Auto-correlation (DMA) for *trans*-NMA (**B,C**) and the right one for *cis*-NMA (**E,F**) compared to the experimental spectrum (black dashed line). Compared are two different machine-learned force fields used in a simulation to obtain the input geometries for the spectra calculation, namely ANI2X (**B,E**) and MACE (**C,F**).
